# Maintenance of high quality of life as an indicator of resilience during COVID-19 social distancing among community-dwelling older adults in Finland

**DOI:** 10.1007/s11136-021-03002-0

**Published:** 2021-09-27

**Authors:** Kaisa Koivunen, Erja Portegijs, Elina Sillanpää, Johanna Eronen, Katja Kokko, Taina Rantanen

**Affiliations:** 1 Gerontology Research Center and Faculty of Sport and Health Sciences, University of Jyväskylä, PO Box 35, 40014 Jyväskylä, Finland; 2 Present Address: Department of Human Movement Sciences, University Medical Center Groningen, University of Groningen, Groningen, The Netherlands; 3 Institute for Molecular Medicine Finland (FIMM), Helsinki, Finland

**Keywords:** Physical function, Psychosocial resources, Adversity, Adaptation

## Abstract

**Purpose:**

Social distancing during the COVID-19 pandemic reduced possibilities for activities of choice potentially threatening quality of life (QoL). We defined QoL resilience as maintaining high quality of life and studied whether walking speed, absence of loneliness, living arrangement, and stress-coping ability predict QoL resilience among older people.

**Methods:**

Community-dwelling 75-, 80-, and 85-year-old persons (*n* = 685) were interviewed and examined in 2017–2018 and were followed up during COVID-19 social distancing in 2020. We assessed QoL using the OPQOL-brief scale and set a cut-off for ‘constant high’ based on staying in the highest baseline quartile over the follow-up and categorized all others as having ‘low/moderate’. Perceived restrictiveness of the social distancing recommendations was examined with one item and was categorized as ‘yes’ or ‘no’ restrictiveness.

**Results:**

Better stress-coping ability (OR 1.21, 95% CI 1.14–1.28) and not being lonely (OR 2.67, 95% CI 1.48–4.63) increased the odds for constant high QoL from before to amid social distancing, and the odds did not differ according to the perceived restrictiveness of the social distancing recommendations. Higher walking speed predicted constant high QoL only among those perceiving restrictiveness (OR 1.16, 95% CI 1.07–1.27). Living arrangement did not predict constant high QoL.

**Conclusion:**

During social distancing, psychosocial resources helped to maintain good QoL regardless how restrictive the social distancing recommendations were perceived to be. Better physical capacity was important for constant high QoL only among those perceiving restrictiveness presumably because it enabled replacing blocked activities with open outdoor physical activities.

## Introduction

Resilience refers to the process of adaptation to or dealing with adversity in a positive way [1, 2]. The manifestation of resilience is likely to vary depending on the adversity, time, life phase and life domain in question. At higher ages, the probability of encountering adversities, such as health decline or social losses increases [3], underlining the relevance of resilience for aging well. Examination of resilience needs to take into account the adversity and the indicator of positive adaptation appropriate in that specific context, as well as the resources that are important for achieving good outcomes despite adversity [2]. The ecological framework of resilience posits that resources promoting resilience may be summoned from three interacting levels of functioning: individual (e.g., psychological resources, physiological reserve), social (e.g., social support) and environmental/structural (e.g., health services and policies) [4].

The relationships between resources, adversity and positive adaptation can be modeled in various ways [5]. For example, Netuveli et al. [6] studied older adult’s resilience longitudinally using a general health questionnaire. Adversity was defined as illness, change in marital status to single, or transition into poverty, and resilience as bouncing back to the pre-adversity mental health level after adversity. In majority of studies among older adults, resilience has been studied as positive psychosocial functioning in the context of accumulating and persisting health adversities such as decreased physical functioning or disability [7, 8], cognitive impairment [9], and caregiver stress [10]. Diversity in operationalizing resilience stems not only from differences in research questions and study designs, but also from challenges related to capturing adaptation processes, as the timing and types of adversities vary between individuals making it challenging to construct analytical models in observational studies.

The social distancing recommendations designed to curb the spread of the SARS-CoV-2 virus causing the COVID-19 disease has created an unforeseen natural experimental setting allowing us to specify an adversity encountered by the whole population at the same time. In Finland, during spring 2020, persons aged 70 years and older were advised to shelter at home, i.e., avoid close physical contacts with other than the immediate members of their household. All social activities were suspended and destinations of interest, such as restaurants, exercise facilities and social clubs, were closed, thereby reducing older adults’ possibilities for social interaction, participation in meaningful activities and community mobility. While functioning to contain the spread of the virus, social distancing simultaneously imposed an intervention that, by reducing social and environmental resources for meaningful activity, influenced negatively on many of the components of older adults’ quality of life (QoL) [11] and psychosocial functioning [12, 13]. Hence, considering the adversity related to COVID-19 social distancing among older adults, we argue that measuring stability and change in QoL may capture important aspects of the dynamics of adaptation.

Previous studies have found that psychosocial resources, such as positive coping behaviors and social support help to sustain well-being amid the COVID-19 pandemic [13, 14]. Especially older adults living in single households may be particularly vulnerable to social isolation and loneliness amid the pandemic [13], increasing the risk for mental and physical health decline [15, 16]. Creese et al. [17] reported that not perceiving oneself lonely and maintaining a higher level of physical activity protected against declining mental health during the pandemic among adults over 50 years. Especially among older people, decreased physical function may reduce their possibilities for salutary activity particularly when environmental support and opportunities are limited [18]. To the best of our knowledge, the role of older adults’ physical functioning predicting resilience during COVID-19 pandemic has not been studied.

## Present study

The objective of this study is to examine factors promoting QoL resilience among older people during a period of social distancing. We apply the ecological resilience framework and define the individual, social and environmental resources, the adversity and the adaptation as follows. We studied stress-coping ability and walking speed as individual resources. In this study, we conceptualized stress-coping as self-reliance in one’s ability to manage with different adversities of life and assessed it using a scale of psychological resilience. Walking speed was used to indicate physiological resources, which measures the ability to move but is also a widely used summary indicator of vitality reflecting various physiological capacities underlying health and the aging process [19, 20]. Consequently, walking speed may indicate a person’s physical reserve for recovery and adaption when encountering adverse events [21]. Potential social resources for resilience were identified with perceived loneliness. Loneliness is a subjective perception of social isolation or lack of connectedness with others. In this study, we conceptualized the absence of loneliness as an indicator of social connectedness and provision. Environmental resources were captured with the living arrangement. Living with someone may provide emotional and practical social support [22], which have been recognized as important resources for resilience in older ages [23]. In our analyses, social distancing recommendation was the adversity that was encountered by all. Our preliminary unpublished analyses suggested that people who had been more active perceived the social distancing recommendation more restrictive, and potentially were at a higher risk of QoL decline. We defined resilience as maintaining high QoL throughout the follow-up period from two years before to amid social distancing when the second assessment was conducted. Our idea was to test the buffering hypothesis, which proposes that specific factors are particularly beneficial in achieving positive outcomes when facing the adversity [24].

### The context of social distancing in Finland

The Finnish government declared a state of emergency caused by the COVID-19 pandemic on 16 March 2020. To protect the population and the healthcare system from the consequences of a highly infectious disease, the Emergency Powers Act was passed. As a result, public gatherings were limited to no more than ten persons and avoiding spending unnecessary time in public places was recommended. All public cultural and social institutions, exercise facilities, clubs, organizations’ social spaces, and other social activities were closed down. Private sector, third sector, and religious communities were advised to do the same. Due to their higher risk for severe infection, people aged 70 and over were recommended to remain at home and to avoid close physical contacts with others outside their household. However, people were encouraged to continue outdoor activities while maintaining the recommended physical distance to others. The state of emergency remained in force in Finland for three months, ending on 16 June 2020. However, people were still advised to continue maintaining a safe physical distance to others. According to recent studies, during the first wave of the pandemic, three quarters of Finnish older adults adopted some distancing practices [25] and older adults over 70 years reported almost 90% fewer physical contacts as compared to normal conditions [26].

## Methods

The present participants were drawn from the ‘Active aging—resilience and external support as modifiers of the disablement outcome’ (AGNES) study [27]. Here, we present longitudinal analyses of the follow-up extending from 2017–2018 (approximately two years before COVID-19 pandemic) to 2020 (amid the COVID-19 social distancing recommendations).

At the baseline, the participants comprised three age cohorts (75, 80, and 85 years) who were living independently in the city of Jyväskylä, Finland, and whose contact information was obtained from the population register of the national Digital and Population Services Agency. At baseline, the exclusion criteria were not living independently in the recruitment area and inability to communicate. Of all the people we contacted to form the baseline sample, 36.6% took part in the study [28]. The baseline sample consists of altogether 1 018 individuals who took part in a computer-assisted personal interview (CAPI) administered in their homes. Details of the protocol, recruitment and participation in the baseline study are reported elsewhere [27, 28].

The surviving 985 baseline participants who had not withdrawn their consent, formed the target group for the AGNES-COVID-19 follow-up survey in 2020. To avoid physical contact, data were collected using a postal questionnaire or by an interview over the phone, if the participant had difficulty answering the questionnaire or preferred an interview. In total, 809 (58% women) responses were received in the follow-up survey. The participation rate (82%) did not differ by sex. Recruitment and participation in the follow-up study are reported in detail elsewhere [11].

The analyses of the present study comprise all the participants for whom both baseline and follow-up data on QoL and all the selected predictors from the baseline were available (*n* = 685; 290 men and 395 women).

### Measurements of resilience

#### Quality of life

Quality of Life (QoL) was assessed with the 13-item version of the Older People’s Quality of Life questionnaire (OPQOL-brief) at baseline and during social distancing. The items included in the scale are related to both life overall and to more specific themes such as health, independence and control over life, social relationships and leisure/social activities, home and neighborhood, psychological and emotional well-being, and financial circumstances. Response options range from one (strongly disagree) to five (strongly agree). The total sum score ranges from 13 to 65 with higher values indicating higher quality of life. The OPQOL-brief has shown to be valid and reliable measurement among older adults [29].

#### Operationalizing QoL resilience

We specified resilience as maintaining constant high QoL despite perceiving social distancing as restrictive. The category of *constant high QoL* was defined as a QoL score in the highest quartile at baseline (≥ 59 points) and maintaining it at the same level during the period of social distancing. Participants who did not meet these criteria were considered to have *low/moderate QoL*.

We considered that the perceived restrictiveness of the social distancing recommendations would indicate how troublesome this specific adversity caused by the COVID-19 situation was for the participant. We asked the participants to assess on a 5-point response scale ranging from zero (not at all) to 4 (very much) the extent to which the social distancing recommendations prevented them from engaging in activities they would have liked to do. The responses “not at all” and “little” were categorized as *NO perceived restrictiveness,* indicating less severe adversity, and the responses “somewhat”, “much” and “very much” were categorized as YES *perceived restrictiveness,* indicating more severe adversity.

For the statistical analyses, we created a variable from different combinations of the categories of QoL and the perceived restrictiveness of the social distancing recommendations as follows: constant high QoL + yes perceived restrictiveness (QoL resilience), constant high QoL + no perceived restrictiveness, low/moderate QoL + yes perceived restrictiveness, and low/moderate QoL + no perceived restrictiveness.

### Assessments of individual and social resources predicting QoL resilience

We assessed self-rated *stress-coping ability* at baseline using the ten-item Connor-Davidson Resilience scale (CD-RISC), which measures the perceived ability to adapt positively to changes in life [30, 31]. The scale includes items such as*”I am able to adapt when changes occur”* and *“I think of myself as a strong person when dealing with life’s challenges and difficulties”*. The response scale ranges from 0 (not true at all) to 4 (true nearly all the time) totaling a sum score, which range from 0 to 40 (a higher score indicating higher self-reliance in ability to cope with adversity). The scale has shown good measurement properties among Finnish older adults in most of the psychometric domains [32]. Our analyses only included participants with at most three missing items in their answers. Scores for missing items were imputed for 12 participants based on the mean of their responses to the other items.

Both *living arrangement* and *loneliness* were asked at baseline. Living arrangement was defined as living alone versus not living alone (a partner or another adult, e.g., family member). Loneliness was measured using a single structured item with four response options: 1 (almost always), 2 (often), 3 (rarely) and 4 (very rarely/never). For statistical analyses, the responses were recoded as *yes, at least sometimes* (“almost always” to “rarely”) and *no* (“very rarely/never”).

Maximal *10-m walking speed* was assessed at baseline in the laboratory corridor using photocells (Faculty of Sport and Health Sciences, University of Jyvaskyla, Jyväskylä, Finland). Participants were instructed to walk as fast as possible, without compromising safety. Five meters were allowed for acceleration. Participants wore walking shoes or sneakers and were allowed to use a walking aid if needed [27].

For the multivariate analyses, we selected potential confounders from the baseline data based on their likely association with the predictors and QoL. These variables included sex, age, cognitive functioning, and chronic conditions. Cognitive functioning was tested with Mini Mental State Examination (MMSE) with higher scores indicating better cognition [33]. Number of chronic conditions was calculated based on responses to a questionnaire and subsequently reviewed by a research nurse [27].

### Statistical analysis

We used paired samples *t* test and linear regression analysis to describe changes in QoL between the baseline measures and those recorded during social distancing. To compare the characteristics of the participants according to the dichotomous categories of QoL and perceived restrictiveness of social distancing, we used independent samples t-test for continuous variables and chi-square test for categorical variables. Subsequently, we studied possible predictors of constant high QoL and high perceived restrictiveness of social distancing separately using logistic regression analysis. The potential predictors were baseline stress-coping ability, absence of loneliness, living arrangement and walking speed. To test whether the associations of significant predictors with constant high QoL vary according to perceived restrictiveness, we ran separate logistic regression analyses for each predictor by adding the interaction term of predictor-by-perceived restrictiveness of social distancing with the main effects in the model. Finally, to identify the predictors of QoL resilience, we used multinomial logistic regression analysis with the nominal combination variable of QoL and perceived restrictiveness of social distancing as an outcome (reference group: low/moderate QoL + no perceived restrictiveness). All the predictors were added in the model simultaneously and the model was adjusted for age, sex, MMSE, education and chronic conditions.

To test the robustness of our findings, we stratified the main analyses according to sex. The results did not change substantially and therefore we report the models for both sexes combined. All analyses were computed using SPSS Statistics 26 for Windows.

## Results

Average QoL at baseline was 55.1 points (SD 5.5) and during social distancing 53.5 points (SD 6.8). The average decline in QoL between the baseline and social distancing measurements was 1.6 points (SD 5.5, *p* < 0.001). The change ranged from a 13-point decrease to a 24-point increase and was not clearly attributable to any single OPQOL-brief items. Linear regression analysis showed that a higher baseline QoL was associated with a higher decline in QoL (*β* − 0.236, *p* < 0.001). In addition, perceived restrictiveness of the social distancing recommendations was associated with a higher decline in QoL (*β* − 1.931, *p* < 0.001). On our definition, 15% of the participants were categorized as having constant high QoL and 85% as having low/moderate QoL. In addition, 63% of the participants were categorized as perceiving restrictiveness owing to the social distancing recommendations.

Table 1 shows the characteristics of the participants according to the two QoL categories and the two categories of the perceived restrictiveness of the social distancing recommendations. On average, the participants with constant high QoL had higher self-rated stress-coping ability and walking speed at baseline compared to those with low/moderate QoL. In addition, those with constant high QoL were younger and reported less loneliness at baseline than those with low/moderate QoL. Perceived restrictiveness of the social distancing recommendations and living arrangement did not differ between the QoL categories.

**Table 1:** Participant characteristics by perceived restrictiveness of social distancing and quality of life (QoL) categories

	QoL		*p*^a^	Perceived restrictiveness of social distancing		*p*^a^
	Constant high (*n* = 104)	Low/moderate (*n* = 581)		Yes (*n* = 432)	No (*n* = 253)	
	*n* (%)	*n* (%)		*n* (%)	*n* (%)	
Perceived restrictiveness of social distancing recommendations, high	60 (58)	372 (64)	.218	**–**	**–**	**–**
QoL, constant high	**–**	**–**	**–**	327 (86)	209 (82)	.218
Living arrangement, alone	37 (36)	226 (39)	.521	170 (39)	93 (37)	.501
Loneliness, no	86 (83)	317 (55)	** < .001**	236 (55)	167 (66)	**.004**
Age						
75 years	63 (61)	281 (48)	**.027**	226 (52)	118 (47)	.337
80 years	31 (30)	190 (33)		135 (31)	86 (34)	
85 years	10 (10)	110 (19)		71 (16)	49 (19)	
Sex, women	71 (53)	324 (59)		276 (64)	119 (47)	** < .001**
	Mean (sd)	Mean (sd)	*p*^b^	Mean (sd)	Mean (sd)	*p*^b^
Stress-coping ability	35.0 (4.1)	31.0 (4.9)	** < .001**	31.1 (4.9)	32.0 (5.1)	**.022**
Walking speed, m/s	1.9 (0.4)	1.8 (0.4)	** < .001**	1.8 (0.4)	1.8 (0.4)	.067
Chronic conditions, number	2.7 (1.8)	3.4 (2.0)	** < .001**	3.5 (2.0)	3.1 (1.9)	**.013**
MMSE	28.0 (1.8)	27.5 (2.1)	**.016**	27.8 (2.0)	27.3 (2.3)	**.009**

Bold typeface indicates statistically significant at the significance level of .05

The category *no perceived restrictiveness of social distancing recommendation* included the responses “not at all” and “little” and the category *yes perceived restrictiveness* included the responses “somewhat”, “much” and “very much”; the criterion for membership of the category *constant high QoL* was a QoL score in the highest quartile at baseline (≥ 59 points) and maintaining it at the same level during social distancing. Participants not meeting this criterion were considered to have *low/moderate QoL*; the category *no loneliness* included the response option “very rarely/never” and the category *loneliness at least sometimes* the response options from “almost always” to “rarely”

^a^Tested with chi-square test

^b^Tested with *t* test

A greater proportion of the participants who perceived the social distancing recommendations as restrictive were women and had on average more chronic conditions than those not perceiving restrictiveness. In addition, the participants perceiving high restrictiveness reported more loneliness, had lower self-rated stress-coping ability, and had slightly higher MMSE scores at baseline than those perceiving no restrictiveness.

The logistic regression analysis showed that higher walking speed and stress-coping ability and the absence of loneliness predicted constant high QoL (Table 2). In addition, the absence of loneliness reduced the likelihood of perceiving the social distancing recommendations as restrictive (OR 0.62, 95% CI 0.45–0.86), whereas stress-coping ability, living arrangement, and walking speed were not associated with the perceived restrictiveness of social distancing recommendations. The separate analyses for the predictors of constant high QoL showed significant interaction of walking speed-by-perceived restrictiveness (*p* = 0.005) indicating a stronger association between walking speed and constant high QoL among participants who perceived the social distancing recommendations to be restrictive. The interactions of loneliness and stress-coping ability by perceived restrictiveness of the social distancing recommendations with constant high QoL were not significant indicating that the associations with constant high QoL in both categories of perceived restrictiveness were similar.

**Table 2:** Odds ratios for constant high (*n* = 104) vs. low/moderate (*n* = 581) QoL and perceived (*n* = 432) vs. no perceived (*n* = 253) restrictiveness of social distancing

	QoL^a^	Perceived restrictiveness of social distancing recommendations^a^
	Constant high (*n* = 104)	Yes (*n* = 432)
	OR (95% CI)	OR (95% CI)
Walking speed, per 0.1 m/s	**1.08 (1.01–1.15)**	0.98 (0.95–1.02)
Stress-coping ability, per 1 point	**1.21 (1.14–1.28)**	0.98 (0.95–1.02)
Loneliness, ‘no’ vs. ‘yes, at least sometimes’	**2.67 (1.48–4.63)**	**0.65 (0.45–0.94)**
Living arrangement, ‘alone’ vs. ‘with someone’	1.34 (0.78–2.30)	0.79 (0.54–1.15)

Bold typeface indicates statistically significant at the significance level of .05

*OR* odds ratio, *CI* confidence interval

^a^Analyzed with logistic regression analysis, constant high vs. low/moderate QoL and yes vs. no perceived restrictiveness of social distancing, adjusted for age, sex, MMSE, education and chronic conditions

The results of the multinomial logistic regression analysis with the combined categories of QoL and perceived restrictiveness of social distancing recommendations as the outcome are given in Table 3. Higher walking speed was associated with constant high QoL only among the participants who perceived restrictiveness. A 0.1 m/s increase in walking speed was associated with 16% greater odds for membership of the category combining constant high QoL and perceived restrictiveness compared to belonging to the category combining low/moderate QoL and perceived restrictiveness.

**Table 3:** Odds ratios for combinations of QoL and perceived restrictiveness of social distancing categories

	QoL + perceived restrictiveness of social distancing recommendations^a^		
	Constant high QoL + YES perceived restrictiveness (*n* = 60)	Constant high QoL + NO perceived restrictiveness (*n* = 44)	Low/moderate QoL + NO perceived restrictiveness (*n* = 204)
	OR (95% CI)	OR (95% CI)	OR (95% CI)
Walking speed, per 0.1 m/s	**1.16 (1.07–1.27)**	1.01 (0.91–1.12)	1.05 (1.00–1.10)
Stress-coping ability, per 1 point	**1.22 (1.13–1.31)**	**1.22 (1.12–1.32)**	1.02 (0.98–1.06)
Loneliness, ‘no’ vs. ‘yes, at least sometimes’	**3.03 (1.42–6.50)**	**3.36 (1.37–8.27)**	**1.57 (1.07–2.31)**
Living arrangement, ‘alone’ vs. ‘with someone’	0.75 (0.35–1.41)	0.58 (0.26–1.29)	0.77 (0.61–1.16)

Bold typeface indicates statistically significant at the significance level of .05

*OR* odds ratio, *CI* confidence interval

^a^Analyzed with multinomial logistic regression analysis, reference group: low/moderate QoL + YES perceived restrictiveness of social distancing, adjusted for age, sex, MMSE, education and chronic conditions

Participants with better stress-coping ability had higher odds for maintaining constant high QoL regardless of how restrictive they perceived the social distancing recommendations to be when compared to those in the category combining low/moderate QoL and perceived restrictiveness. The absence of loneliness was associated with one and a half greater odds for having low/moderate QoL + no perceived restrictiveness when compared to those having low/moderate QoL + perceived restrictiveness. In addition, the absence of loneliness was associated with over threefold odds for maintaining constant high QoL in both groups of perceived restrictiveness. Finally, living arrangement was not associated with maintaining constant high QoL.

## Discussion

Our analyses showed that higher self-rated stress-coping ability and the absence of loneliness predicted the maintenance of high QOL similarly among those who perceived or did not perceive social distancing as restrictive. Higher walking speed was an important predictor of maintenance of high QoL only among those who perceived social distancing as restricting their activities. Finally, living arrangement was not associated with the maintenance of high QoL. The present analyses yield important insights into the adaptive processes that older adults used in the specific context of the social distancing recommendations. The study also contributes to understanding resilience in later life, which has been recognized as an important element in aging well [34].

An association between higher walking speed and good QoL in older adults has been reported earlier [35]. To the best of our knowledge, the present study is the first to suggest that walking speed indicating physical resources may be particularly beneficial in the maintenance of good QoL when facing vs. not facing adversities. The current finding coheres with our previous study showing that walking speed was a stronger predictor of survival among participants who sustained versus did not sustain a bone fracture [21]. In the present as in the previous study, the mechanism underlying the effect of walking speed kicking in the presence of adversity and buffering the negative effects probably lies in the availability of a functional reserve that the individual can tap into. In addition to capturing health and the aging process [20], higher walking speed indicates a better capacity for all bodily movement, especially the ability to move in one’s environment [19], an important resource for autonomy and meaningful activities. When access to environmental activity resources, for example, exercise facilities and social clubs, was reduced by the social distancing recommendations, older adults with a better ability to move may have had a higher readiness to substitute their suspended activities of choice with alternatives that were not blocked. According to our recent report, most of the activities reported by our participants during the studied period of recommended social distancing encompassed walking for fitness and visiting outdoor exercise facilities that remained open [36]. Maintaining a desired activity level by increasing outdoor exercise may have bestowed a sense of continuity while also manifesting further advantages, such as improved fitness [37] and restorative experiences [38]. We also recently reported that those who did not perceive the social distancing recommendations as restrictive were less likely to change their physical activity behavior [39]. Many older people prefer activities that take place at home (e.g., crafting, DIY, gardening). Such activities were not affected by the social distancing recommendations, and consequently people inclining to them did not need to draw on extra individual functional resources to maintain their QoL.

The associations of the absence of loneliness and better stress-coping ability with the maintenance of high QoL are in accordance with previous findings [40–42]. However, our results suggest that in the studied context, these factors did not have a buffering effect but were generally important for all maintaining good QoL. This may be explained by the nature of these variables in relation to the perceived restrictiveness of social distancing recommendations as the adversity. Loneliness was measured with one item, which has been shown to correlate especially with the emotional dimension of loneliness that springs from the longing for close emotional attachment figure rather than social loneliness, which arises from the absence of an engaging social network [43, 44]. In addition, self-rated stress-coping ability, as it was measured in this study, may reflect an overall optimistic view of one’s personal agency in adversities general [45]. The finding that living arrangement was not associated with maintenance of high QoL is consistent with some earlier studies showing that older adults living in single households, at least in Western cultures, are not necessarily more vulnerable than individuals living with another person [46–48].

In this study, our approach in operationalizing resilience aligns with the individual-centered method using researcher-driven distribution-based thresholds [5]. This allowed us to take into account the relationship between the perception of the adversity and the outcome, and to identify a conceptually meaningful subgroup of individuals assumed to show resilience. However, a major disadvantage is the absence of any established thresholds that can be applied in defining resilience. Thus, because no standardized cut-off value for high QoL exists in the OPQOL-brief scale, we set the threshold for high QoL based on the distribution in the baseline QoL. We decided to set the threshold high in the distribution (the upper quartile of baseline QoL) as we assumed that in its severity, the social distancing recommendations is a moderate rather than catastrophic or traumatic type of adversity [1]. However, this approach may capture only a small subset of people adapting well or showing robustness in adversity, and other approaches quantifying resilience should also be explored. Future studies could investigate QoL trajectories after social distancing recommendations have ended to find out whether people with decreased QoL bounce back to their initial level of QoL after the normalization of environmental opportunities for their preferred activities.

This study has its limitations. One is that we cannot rule out other possible reasons, such as aging or other individual adversities, which may have contributed to changes in QoL. However, in our sample, QoL did not change during a one year follow-up observed before social distancing [49] and in the present analyses, the perceived restrictiveness of the social distancing recommendations was associated with higher decline in QoL supporting our premise that the social distancing measures constituted a natural experimental setting threatening QoL. Another limitation was that due to the social distancing recommendations, the data collection method at baseline (CAPI) differed from that used at follow-up (postal questionnaire), which may have biased the results. However, the follow-up questionnaires were carefully filled in and contained only little missing information. Selection bias may also have occurred, as our participants represented a slightly healthier section of the same-age population [11]. Nevertheless, the participants ranged widely in many background characteristics.

The major strength of this study was that we were able to operationalize resilience in a context of adversity (social distancing measures) that applied to all people at the same time. Opportunities to standardize an adversity are rare in resilience research, especially in a population-based representative and heterogeneous sample instead of a self-selected convenience sample. Our approach has most likely helped to minimize the possibility of bias. Finally, the baseline data collected two years before social distancing included a wide range of information on the participants’ functioning and enabled us to study longitudinally pre-adversity resources in different life domains as predictors of adaptation. While most research has focused to investigate the psychosocial resources for resilience during the pandemic, this study extends the findings on the importance of physical resources for older adults’ adaptation.

Taken together, the findings indicate that in the context of social distancing recommendations, psychosocial resources were important for maintaining good QoL regardless of how restrictive social distancing was perceived. Higher physical resources, in turn, were important among those perceiving restrictiveness, as they possibly enabled adaptive strategies to engage in alternative activities of choice and consequently, to maintain good QoL despite environmental restrictions. These findings highlight the importance of recognizing older adults’ resources across multiple levels of functioning to adapt during challenging times, such as the ongoing pandemic.
